# Practice of Monitoring Cisplatin-Induced Ototoxicity by Audiology, ENT, and Oncology Specialists: A Survey-Based Study in a Single Italian Medical Center

**DOI:** 10.3390/audiolres13050069

**Published:** 2023-10-18

**Authors:** Valeria Gambacorta, Eva Orzan, Mario Faralli, Mario Gullà, Ruggero Lapenna, Irene Baietta, Verena De Angelis, Giampietro Ricci

**Affiliations:** 1 Department of Medicine & Surgery, Section of Otorhinolaryngology, University of Perugia, 06126 Perugia, Italy; gambacortavaleria@gmail.com (V.G.);; 2 Audiology and Otorhinolaryngology Unit, Institute for Maternal and Child Health IRCCS “Burlo Garofolo”, 34137 Trieste, Italy; 3 Clinical Oncology, Santa Maria della Misericordia Hospital, University of Perugia, 06126 Perugia, Italy

**Keywords:** ototoxicity, hearing impairment, cisplatin, tinnitus, ototoxicity monitoring

## Abstract

Ototoxic drugs can result in hearing loss and tinnitus. Early detection of the ototoxic process can help minimize or prevent these consequences. The American Speech–Language–Hearing Association has provided guidelines for monitoring ototoxicity, whereas Italy has not yet implemented a national monitoring protocol. This study aims to assess the current state of ototoxicity monitoring in patients receiving cisplatin therapy. A self-administered survey has been used to gather information from oncologists, audiologists, and ENT specialists. The research was conducted at Santa Maria della Misericordia hospital in Perugia. Two questionnaires were administered, one to ENT/audiology specialists and another to oncology specialists. Both questionnaires were used to collect information on awareness of chemotherapy-induced ototoxicity. A comprehensive understanding of cisplatin-induced ototoxicity has been widely established (100%). The most commonly reported audiological symptoms by patients were hearing loss (100%) and tinnitus (87.5%). The majority of ENT and audiologists (93.8%) and oncologists (92.9%) expressed the need for a specific ototoxic monitoring program. However, they noted the absence of a well-defined ototoxicity monitoring protocol. A well-established and efficient ototoxic monitoring system facilitates early detection of ototoxic hearing loss and subsequent rehabilitation of inevitable hearing impairment.

## 1. Introduction

Ototoxicity refers to the dysfunction of the auditory-vestibular system, which can be temporary or permanent. This dysfunction can be induced by the use of specific medications, exposure to chemicals, and ionizing radiation [1]. The etiology of cisplatin-induced hearing loss can be attributed to alterations in the cochlea. Specifically, the initial occurrence involves the deterioration of the outer hair cells, followed by the subsequent impairment of the inner hair cells, progressing in order from the basal region (associated with high frequencies) to the apical region (associated with low frequencies). In addition to the aforementioned, the supporting cells, spiral ganglion cells, and marginal cells of the stria vascularis are also subject to impairment.

The degree of hearing loss can be attributed to several factors, including the individual’s prior exposure to loud noises, the length of their therapy, and the total amount of platinum-based medications they have received. The mechanisms of ototoxicity are currently understood to be associated with the generation of free radicals. The literature review also emphasizes the presence of a genetic component in the risk factors associated with cisplatin-induced ototoxicity [2]. The occurrence of ototoxicity in the peripheral vestibular system is associated with either the deafferentation of the vestibular end organs or the partial or complete loss of hair cells. The manifestation of bilateral loss of the vestibular system is characterized by postural instability and oscillopsia. These symptoms occur when there is a lack of information from the other two sensory systems, somesthesia and vision, due to their absence or alteration caused by the disease [3]. The potential repercussions of ototoxic drugs, such as permanent hearing loss or balance disorders, can significantly impact individuals in their occupational, educational, and social domains. The mitigation or avoidance of these consequences can be achieved through early detection of the ototoxic process during treatment.

The primary objective of a proficient monitoring program is to identify ototoxic impairment prior to the patient’s manifestation of symptoms. The timely identification of a medical condition enables healthcare professionals to explore various treatment options, such as adjusting the dosage of a medication or transitioning to a less harmful pharmaceutical, in order to impede or cease the advancement of inner ear impairment. In case of permanent sensorineural hearing loss, the utilization of hearing aids or cochlear implants is employed to reduce the extent of the disability [4]. Monitoring for ototoxicity is not widely practiced and there is inconsistency in the procedures used for testing. This is primarily due to the lack of universally agreed-upon guidelines for selecting patients, determining schedules and monitoring times, and interpreting test results.

There are currently more than 600 drugs that are classified as ototoxic. These include platinum-based chemotherapy agents (such as cisplatin and carboplatin), aminoglycoside antibiotics, loop diuretics, macrolide antibiotics, and antimalarial and antiretroviral drugs [5,6,7,8]. Platinum-based agents are utilized in the treatment of various malignancies across different age groups, including both pediatric and adult populations [9]. Cisplatin is recognized for its ototoxicity, with reported incidence rates of approximately 50–80% in adults and 60–90% in children. It commonly results in bilateral sensorineural hearing loss, initially affecting high frequencies (9–20 kHz) and gradually extending to lower frequencies with continued exposure [10]. Permanent bilateral sensorineural hearing loss and tinnitus can manifest during treatment and up to 136 months following therapy, with an incidence ranging from 20% to 84% [11]. Vestibular toxicity is rare, thus vestibular examination is not commonly conducted in patients receiving ototoxic therapy. Several tests can be used to assess vestibular function, including vestibular-evoked myogenic potentials (VEMPs), a video head impulse test (vHIT), and videonystagmography (VNG). These tests can be complemented by bedside examination and subjective questionnaires [12,13].

The American Speech–Language–Hearing Association (ASHA) recommends the implementation of an auditory cochleotoxicity monitoring protocol. This protocol should involve collaboration between audiologists, oncologists, and other healthcare professionals who treat patients receiving ototoxic drugs. The optimal test schedule is primarily determined by the patient’s medication regimen [14]. ASHA and the American Academy of Audiology recommend that baseline assessment should include objective measures like otoacoustic emissions (OAEs) and tympanometry, as well as subjective measurements like pure-tone audiometry (PTA) from 250 Hz to 8000 Hz and high-frequency audiometry (HFA) from 9000 Hz to 20,000 Hz, as well as self-evaluation questionnaires. The utility, reliability, and application of these techniques varies depending on the patient characteristics and patient collaboration, and they can also be utilized in combination [14,15]. According to the ASHA, the baseline test for patients receiving cisplatin-based chemotherapy should be performed no later than 24 h after the initial dose and no later than 1 week beforehand. Thereafter, testing should be conducted no later than 24 h before each subsequent cycle of treatment. Within 24 h, the patient should be retested to confirm the injury if a drop in hearing threshold is noted. In order to assess any long-term residual effects, additional follow-up actions include audiological testing right after treatment as well as 3 months, 6 months, and 1 year later [14]. In spite of global forecasts predicting a 47% increase by 2040, Italy has not yet implemented a national monitoring procedure to identify and monitor ototoxicity in chemotherapy patients [16].

This study aims to evaluate the current status of ototoxicity monitoring in patients receiving cisplatin therapy by utilizing a self-administered survey for oncologists, audiologists, and ENT specialists, as research in this area is currently limited.

## 2. Materials and Methods

The study was carried out at Santa Maria della Misericordia hospital in Perugia. Two separate questionnaires were administered to specialists in the fields of ENT and audiology, as well as oncology, within the Umbria region. The oncology specialists participating in this study provide medical care to patients afflicted with various forms of neoplastic diseases, with a specific focus on the management of lung, breast, gastrointestinal, gynecological, and genitourinary carcinomas. Two questionnaires were used to gather information on awareness of chemotherapy-associated ototoxicity, patient-reported symptoms, perceived roles and responsibilities of specialists, and current practices in identifying and managing patients at risk for cisplatin-associated ototoxicity.

The questionnaires were developed in accordance with the guidelines provided by ASHA [14] and the American Academy of Audiology (AAA) [15] for managing patients who are taking ototoxic drugs. The reports were made using Google Forms, a survey administration software (Version number 0.8) that is a component of the free Google Docs Editors suite offered by Google. The hypertext link containing the survey was emailed to the target population for completion and submission through Google Forms.

### 2.1. ENTs/Audiologists’ Survey

The survey comprised 19 questions that explored several aspects (Table 1).

Demographics section: the clinical role of the respondent and the specific patient population they treat, such as adults, children, or both;

Knowledge section: specialist’s knowledge of drug-induced ototoxicity;

Management section: which specialist is responsible for providing information regarding ototoxic effects;

Medical history section: audiological symptoms patient reports and audiological history;

Monitoring program section: examines the existence of an ototoxicity monitoring protocol in the department, the importance of a multidisciplinary team, and the required tests for audiological assessment.

### 2.2. Oncologists’ Survey

The survey comprised 22 questions, with each section exploring different aspects (Table 2).

Demographics section: the clinical role of the respondent and the specific patient population they treat, such as adults, children, or both;

Management section: the number of patients referred for audiological assessment;

Medical history section: audiological symptoms reported by patients and audiological anamnesis;

Knowledge section: expertise in drug-induced ototoxicity;

Management section: reference figure to whom patients undergoing ototoxic therapy refer and a specialist responsible for providing information on ototoxic effects;

Monitoring program section: the importance of a multidisciplinary team and the presence or absence of a monitoring protocol for ototoxicity in the department of affiliation.

### 2.3. Statistical Analysis

Descriptive methods of analysis were employed to analyze the data collected in this study.

## 3. Results

This study involved 60 specialist doctors, comprising 28 oncologists and 32 ENTs and audiologists.

### 3.1. ENTs/Audiologists’ Survey

**Figure 1:**
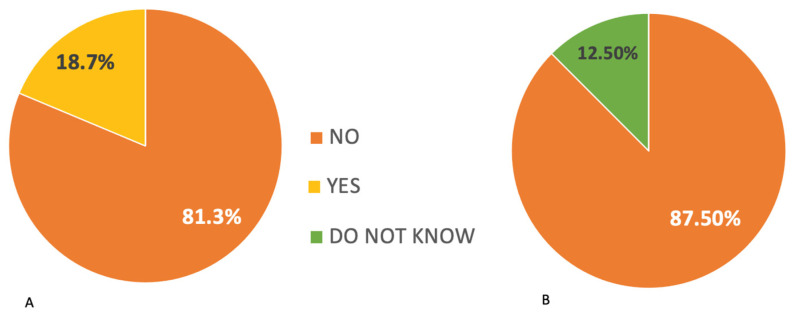
(**A**) Responses of ENTs/audiologists regarding the existence of ototoxicity monitoring in the department where they work. (**B**) Responses of ENTs/audiologists regarding the existence of a protocol for ototoxicity monitoring in the department where they work.

This study found that 81.3% of participants have experience treating both adult and pediatric patients, while 12.5% exclusively treat adult patients and the remaining 6.3% exclusively treat pediatric patients. A comprehensive (100%) understanding of cisplatin-related ototoxicity has been widely established, with cisplatin being the most frequently mentioned drug associated with ototoxicity, followed by amino-glycosides. Based on the responses received, 81.3% of ENTs/audiologists believe that oncologists are the most appropriate healthcare professionals to inform patients about the potential ototoxic effects of chemotherapy medications. The most commonly reported audiological symptoms among patients are hearing loss, which has a prevalence of 100%, and tinnitus, which has a prevalence of 87.5%. The majority of participants (93.8%) expressed the need for a precise ototoxic monitoring program. Among ENTs/audiologists, 81.3% reported the absence of ototoxic monitoring procedures in their department. However, they also noted the absence of a specific protocol outlining the timeline for conducting evaluations in patients undergoing ototoxic chemotherapy (Figure 1).

Otolaryngologists and audiologists recommend conducting specific tests on patients receiving ototoxic drug treatment. The primary test suggested is pure-tone audiometry (100%), followed by tympanogram and cochleo-stapedial reflex tests (43.8%), and finally evoked otoacoustic emissions are recommended by 37.5%.

**Figure 2:**
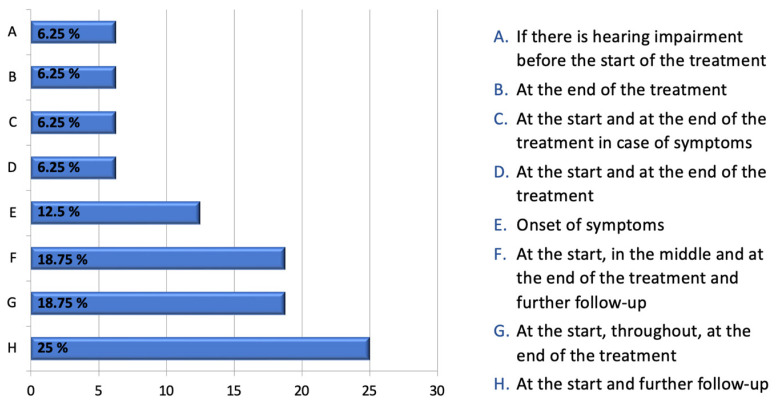
ENTs’ responses to the following question, expressed as a percentage: “If there is no specific protocol for monitoring ototoxicity, when do you think it is appropriate to have an audiological evaluation performed on a patient being treated with chemotherapy drugs?”.

Figure 2 presents a comprehensive overview of the survey results obtained from ENT/audiologists pertaining to the practice of audiometric monitoring. In response to an open-ended question, the specialists indicated what, in their opinion, would be the optimal timing for instituting ototoxicity monitoring. The survey results indicate a widespread consensus regarding the necessity of conducting a baseline audiological test prior to or at the commencement of treatment.

81.3% of respondents advocate for the inclusion of either an audiologist or an otolaryngologist (ENT) in a multidisciplinary team responsible for patients undergoing ototoxic drug treatment. However, the current participation rate of these professionals in such teams stands at only 43.8%.

### 3.2. Oncologists’ Survey

The majority of participants (84.6%) primarily treat adult patients, while a smaller proportion (7.7%) treat both adult and pediatric patients. The remaining 7.7% exclusively treat pediatric patients. All oncologists surveyed (100%) reported awareness of the ototoxic effects of certain chemotherapy drugs.

The symptoms commonly reported by patients, as indicated by oncologists, include hearing impairment (85.7%) and tinnitus (57.1%).

A full awareness of the ototoxicity associated with the use of chemotherapy drugs has been established among all participants (100%). Cisplatin, along with other platinum derivatives, is widely recognized as a leading cause of ototoxic effects.

The majority of oncologists (85.7%) inform patients about the potential ototoxic drug effects prior to initiating chemotherapy. Additionally, all oncologists surveyed believe that they are the most appropriate individuals to provide information regarding the ototoxic effects of drugs.

The majority (71.4%) of oncologist colleagues concur on the value of including an ENT/audiologist specialist in the multidisciplinary team for patients who are eligible for ototoxic drugs.

A large percentage (92.9%) of oncologists advocate for the implementation of a precise ototoxic monitoring program, mentioning a complete absence of such programs in all cases within their respective wards. Despite the evident advantages of implementing a monitoring program, a significant majority of oncologists (92.9%) reported referring less than 20 patients to audiologists in the preceding year.

## 4. Discussion

Cisplatin chemotherapy can cause ototoxic side effects such as hearing loss and tinnitus, which can significantly impact a patient’s social, educational, and occupational well-being. An efficient ototoxic monitoring procedure can identify inner ear damage before a patient experiences subjective impairments in their quality of life. This early detection enables timely intervention to prevent further progression of the injury.

Various strategies can be employed to improve the quality of life for patients experiencing ototoxicity. There are several interventions that can be used to address hearing impairments, including the use of hearing aids or cochlear implants. Speech therapy can also be beneficial. Additionally, modifying the drug or its dosage may be considered. If the patient experiences dizziness, it is likely that vestibular damage has already occurred, and therefore, vestibular rehabilitation is recommended. The American Speech–Language–Hearing Association [14] established guidelines in 1994 for monitoring ototoxicity in patients undergoing treatments. The American Academy of Audiology [15] published a paper in 2009 that presented their stance and provided clinical practice guidelines on the necessity and execution of ototoxic monitoring. These professional organizations recommend conducting comprehensive baseline testing, follow-up assessments before each cycle of platinum-based chemotherapy, and an audiometry follow-up after completing the treatment.

### 4.1. Current Monitoring Protocol

There is currently a lack of recommendations or established guidelines in Italy regarding surveillance during ototoxic chemotherapy treatment. This absence of defined monitoring procedures often leads to patients being referred for audiological evaluation only after symptoms of the injury have already occurred, rather than following a systematic approach. These data emerge also from our survey conducted among oncologists; it reveals that despite acknowledging the benefits of an ototoxic monitoring program, 92.9% of the respondents reported sending fewer than 20 patients for evaluation in the preceding year. These data must be evaluated, taking into consideration that based on the hospital records, a total of 132 individuals underwent treatment with cisplatin within the preceding 12-month period. In contrast, the number of patients assessed in the otolaryngology department within the past year who had cisplatin treatment amounted to a mere eight individuals. The audiological evaluation was requested in these eight cases, subsequent to the patients’ self-reporting of hearing alterations. This observation confirms the prevailing practice wherein patients are assessed by ENT/audiologist specialists solely in response to the emergence of audiological issues, rather than undergoing a hearing test prior to commencing cisplatin therapy. Recent studies have emphasized the inadequate adherence to national monitoring guidelines in various states, indicating that the development and promotion of an effective ototoxic monitoring program requires improved training and collaboration among ENTs, oncologists, and audiologists. This issue extends beyond Italy. A study conducted in the UK revealed that only 28% of participants reported having an ototoxic management protocol in their center, while the remaining 72% reported having none. This finding emphasizes the necessity of implementing standardized management protocols across the UK [4]. Based on previous research undertaken in New Zealand and the United States, it has been observed that there is currently no universally recognized program for monitoring ototoxicity at a national level. According to the findings of the ototoxicity questionnaire administered to specialists, it was shown that they lacked knowledge about the existence of a monitoring program within their services. They mentioned that baseline examinations are only carried out in response to patient-reported hearing disturbances [17]. The findings of our survey align with the existing research, suggesting that a substantial majority (93.8%) of ENT/audiologists understand the importance of instituting a meticulous monitoring program. Moreover, a significant proportion (81.3%) of participants indicated the lack of ototoxicity monitoring protocols. It is imperative to acknowledge the lack of a clearly defined protocol that delineates the optimal timing for conducting audiological examinations.

In this study, it was found that 92.9% of the participating oncologists expressed the importance of implementing a precise ototoxic monitoring program. Furthermore, all cases (100%) within their respective departments reported a lack of such program. The disparity observed between the two categories of specialists can be attributed to the heightened focus of ENTs/audiologists, who, due to their direct involvement with the ear, exhibit a greater level of attentiveness towards the patient’s hearing health.

### 4.2. Role of Specialists

The American Speech–Language–Hearing Association (ASHA) [14] and the American Academy of Audiology (AAA) [15] assert that the audiologist plays a central role in the formulation and implementation of monitoring programs. In light of this rationale, Konrad et al. advocate the inclusion of audiologists within multidisciplinary clinical teams, with the aim of fostering close collaboration to develop optimal care strategies tailored to the unique needs of each patient [18]. According to a study conducted by Paken et al., all oncologists, 78% of nurses, and 69% of pharmacists perceive audiologists as integral members of the cancer patient’s management team. All audiologists confirmed that the hospital lacks a group approach for managing adult patients receiving cancer chemotherapy [19]. The survey results indicate that there is a widely held belief in the significance of including an ENT/audiologist in a multidisciplinary team, even at a local level. Specifically, 71.4% of oncologists and 81.3% of ENTs/audiologists expressed this belief. However, it is worth noting that only 43.8% of ENTs/audiologists reported being part of a multidisciplinary team. Our study found that the majority of ENTs/audiologists (81.3%) and all oncologists believe that the oncologist is the most appropriate healthcare professional to inform patients about the potential ototoxic effects of chemotherapy drugs. These data align with the ASHA guidance, which suggests that it should include more comprehensive information on the signs and symptoms [14]. A survey conducted in South Africa highlighted the necessity of educating oncologists about ototoxicity and its impact on the quality of life of cancer survivors [20].

### 4.3. Timing for Ototoxicity Monitoring

Regarding the timing of ototoxic monitoring, our study highlights that specific guidelines are not followed in Italy and other European countries, such as the United Kingdom. The responses from ENTs/audiologists regarding possible timing in the open-ended question varied.

The prevailing belief was that conducting a hearing examination either at time 0, prior to starting therapy, or at the outset of therapy, was most commonly shared. Cisplatin treatment can lead to cochleotoxicity even after a single course [21], therefore the ASHA emphasizes the significance of obtaining baseline measurements prior to the initial cisplatin dose, or within 24 h after the first administration at the latest [14].

In our study, seven ENTs/audiologists agreed that it is important to conduct hearing tests at the end of treatment. However, none of them recognized the necessity of conducting these tests remotely. It is worth nothing that the literature indicates the possibility of bilateral sensorineural hearing loss occurring even up to 136 months after completing cisplatin therapy [11]. In our opinion, the ASHA guidelines are the most comprehensive as they take into account both the immediate ototoxicity of cisplatin and the potential long-term damage to the inner ear.

### 4.4. Audiological Tests

A specific section of the questionnaire designed for ENTs/audiologists pertains to the audiological tests that are to be conducted. The required examinations consist of tonal audiometry in all instances (100%), followed by tympanogram (43.8%) and cochleostapedial reflexes (43.8%), and lastly, voice audiometry (37.5%) and transient-evoked otoacoustic emissions (TEOAEs—37.5%). Previous research has shown that the use of high-frequency audiometry in prospective investigations has revealed that cochleotoxicity can occur initially or exclusively in the high-frequency range (9–20 kHz [8]). Consequently, incorporating high frequencies into monitoring programs may facilitate the early detection of cochleotoxicity. The ASHA and the AAA recommend the use of high-frequency audiometry as the preferred approach for early identification of ototoxic-induced hearing impairment. Indeed, the initial manifestation of ototoxic drug-induced effects, such as cisplatin, typically occurs at the baseline tonotopic level. Thus, high-frequency audiometry (HFA) enables the identification of hearing impairments well in advance of their discernible presence in traditional audiometric assessments. The standard audiologic assessment, typically limited to frequencies below 8 kHz, does not facilitate the timely identification of ototoxic consequences [14,15]. Regarding TEOAEs, they have demonstrated encouraging outcomes in individuals undergoing cisplatin treatment [22].

Although high-frequency audiometry (HFA) and otoacoustic emissions (OAE) can identify changes prior to the detection of alterations in hearing thresholds across the frequency range spanning from 200 to 8000, HFA generally recognizes ototoxic changes prior to OAEs. OAEs remain valuable in ototoxicity monitoring programs due to their time efficiency and independence from patient cooperation. DPOAEs have the potential to be more sensitive to the first-affected cochlear frequency regions and can detect ototoxic changes earlier than TEOAEs, as they can be monitored at higher frequencies [23]. The Auditory Brainstem Response (ABR) test has limitations regarding administration time and frequency specificity (limited to 1–4 kHz). However, the use of high-frequency tone-burst stimuli may be beneficial as an objective monitoring tool for early ototoxicity diagnosis [24]. It is imperative to emphasize an additional aspect: apart from determining the most appropriate instruments for the development of a clinical protocol, it is essential to consider variables such as the patient’s age, comorbidities, level of cooperation, and therapeutic objectives.

### 4.5. Vestibulotoxicity

While hearing loss is a common condition with well-understood mechanisms, there is currently a lack of established criteria for vestibulotoxicity. Various methods can be employed to assess the function of the vestibular system; the diagnostic methods for identifying bilateral peripheral vestibular system impairment include vestibular-evoked myogenic potentials (VEMPs), rotational testing (vestibular autorotation testing or VAT), caloric testing (bithermal, monothermal warm, or ice water caloric testing), and bedside examination. The head-thrust and dynamic visual acuity tests are not suitable for detecting the initial signs of bilateral peripheral vestibular system impairment that affect lower frequencies. These tests primarily assess high-frequency function and are not sensitive enough for this purpose [3]. In addition to the aforementioned tests, it may be beneficial to administer the Dizziness Handicap Inventory (DHI) questionnaire [25]. The DHI is a self-administered questionnaire that is simple, non-invasive, cost-effective, and efficient for screening purposes. Incorporating DHI into a comprehensive ototoxicity program and conducting a thorough evaluation of its efficacy would be highly beneficial. There is currently no universally agreed-upon standard of care for monitoring vestibulotoxicity, nor is there an established test battery that is suitable for patients who are often seriously ill and difficult to transport.

### 4.6. Our Ototoxicity Monitoring Program

Baseline assessments should be performed for patients receiving cisplatin, ideally within one week prior to the initial treatment. Patients should be re-evaluated at the conclusion of each therapeutic cycle, as well as 3 and 6 months post-treatment to assess any potential long-term residual effects of the drug therapy. It is advisable to perform a follow-up test within 24 h to confirm the initial findings in the case of detecting hearing loss. Cochlear toxicity symptoms necessitate the repetition of tests. The baseline assessment should consist of bilateral pure-tone air conduction thresholds measured at audiometric frequencies ranging from 0.25 to 8 kHz. If it is possible, it is recommended to assess thresholds above 8 kHz. In fact, it would be advantageous to evaluate the frequencies of 9, 10, 11, 12, 14, 16, 18, and 20 kHz. Some patients may not respond to these frequencies, but it is important to document this lack of response during the initial assessment. The initial assessment should consist of a thorough patient case history, including pertinent audiological and vestibular details, along with an otoscopic examination, immittance testing, bone conduction testing, and TEOAE. Speech audiometry, bone conduction, and immittance testing are recommended when hearing loss is observed. The selection of audiological tests for children depends on their age and level of cooperation, necessitating the involvement of a team of ENT/audiologist specialists to choose the appropriate tests for each individual case.

### 4.7. Limitations of the Study

This study is limited by its reliance on Google forms. Estimating the number of individuals who deleted the received email is challenging, and it is difficult to determine the reasons behind their actions, such as lack of interest, time constraints, or perceived irrelevance to their work. For this reason, this study concentrated on a specific institution where it was feasible to individually communicate with all the experts.

Patient-specific data were not collected in this study due to its focus on gathering professionals’ opinions rather than assessing the patients’ status. The aim of this study was to collect data on healthcare awareness regarding the importance of audiological monitoring. Our institution is currently conducting a study on audiological monitoring of cancer patients. We are following the timing and testing suggestions that arise from this study. We will provide detailed information on the patient cohort, including audiological data, tumor type and location, and treatment dosage, once the study progresses.

## 5. Conclusions

This study is the first in Italy to evaluate the implementation of an audiologic monitoring program for patients receiving cisplatin therapy. To ensure widespread acceptance and utilization, an audiologic monitoring program should incorporate efficient and cost-effective research methods that consider the healthcare system and demographics of the patient population to be managed. Close collaboration between otolaryngologists/audiologists and oncologists is essential for successful compliance. This can be achieved by establishing multidisciplinary teams. A well-established ototoxic monitoring system promotes a proactive approach to hearing health by facilitating early detection and rehabilitation of ototoxic hearing loss.

It is essential to carefully select appropriate tests to assess cochleotoxicity, with a preference for high-frequency tests that are more sensitive in detecting early cochlear damage. It is equally essential to prioritize the assessment of vestibulotoxicity and the selection of appropriate test batteries. Although vestibulotoxicity is a less common side effect of ototoxic drugs, it significantly impairs quality of life and requires immediate treatment.

The present study lays the groundwork for establishing a comprehensive ototoxicity monitoring program in Italy. In line with other countries that have successfully implemented such programs, this initiative aims to improve patients’ quality of life concerning ototoxicity and other significant comorbidities.

## Figures and Tables

**Table 1:** Survey questions for ENTs and Audiologists.

Number	Question
1	How long have you been practicing as an ENT specialist/Audiologist?
2	As part of your profession, you deal with? (age of patients)
3	Do you know that some chemotherapy drugs are ototoxic?
4	If you answered yes to the previous question, could you tell us which chemotherapy drugs have an ototoxic effect?
5	In your opinion, who should be entrusted with the task of providing the patient with information regarding the possible ototoxic effect of chemotherapy drugs?
6	In the event of reported audiological symptoms, what symptoms do patients receiving chemotherapy complain about?
7	Do you think that the ENT specialist/Audiologist should be part of a multidisciplinary team caring for patients with neoplastic disease and candidates for potentially ototoxic drugs?
8	Are you part of a multidisciplinary team that treats patients with neoplastic disease?
9	In the anamnesis, do you ask the patient for information about any hearing problems?
10	Do you ask the patient if there is a family history of hearing problems?
11	In which of the following pathologies do you ask the patient for information on taking pharmacological therapy?
12	Do you think having an accurate ototoxicity monitoring program is beneficial?
13	Does the department where you work have procedures for monitoring ototoxicity?
14	If yes, to which patients are they applied?
15	Does the department where you work have a protocol that indicates when the hearing of a patient undergoing ototoxic chemotherapy should be monitored?
16	If you answered yes to the previous question, when is the patient sent for an audiological evaluation?
17	What tests are performed in the event of a monitoring protocol or request from the oncologist specialist?
18	If there is no specific protocol for monitoring ototoxicity, when do you think it is appropriate to have an audiological evaluation performed on a patient being treated with chemotherapy drugs?
19	What audiometric tests do you think are necessary for monitoring ototoxicity?

**Table 2:** Survey questions for Oncologists.

Number	Question
1	As part of your profession, you deal with? (age of patients)
2	How long have you been working with patients suffering from neoplastic disease?
3	How many patients have you referred for an audiological evaluation in the last year?
4	Do patients undergoing chemotherapy complain of audiological symptoms?
5	In the event of reported audiological symptoms, what symptoms do patients complain about?
6	If a patient reports audiological symptoms, to which professional figure is he referred?
7	Do you know that some chemotherapy drugs are ototoxic?
8	If you answered yes to the previous question, could you tell us which chemotherapy drugs have an ototoxic effect?
9	How many patients have you treated with cisplatin-based drugs in the last year?
10	Do you provide patients with information about possible ototoxic effects of drugs before starting chemotherapy?
11	Do you provide the patient with specific recommendations regarding their hearing?
12	If yes, which ones?
13	In your opinion, who should be entrusted with the task of providing the patient with information regarding the possible ototoxic effect of chemotherapy drugs?
14	Do you think that the ENT specialist/Audiologist should be part of a multidisciplinary team caring for patients with neoplastic disease and candidates for potentially ototoxic drugs?
15	In the anamnesis, do you ask the patient information about any hearing problems?
16	Do tou ask the patient if there is a family history of hearing problems?
17	In which of the following pathologies do you ask the patient for information on taking pharmacological therapy?
18	Do you think having an accurate ototoxicity monitoring program is beneficial?
19	Does the department where you work have procedures for monitoring ototoxicity?
20	If you answered yes to the previous question, to which patients are they applied?
21	Does the department where you work have a protocol that indicates when the hearing of a patient undergoing ototoxic chemotherapy should be monitored?
22	If you answered yes to the previous question, when is the patient sent for an audiological evaluation?

## Data Availability

The data presented in this study are available on request from the corresponding author.
